# Chimpanzee choice rates in competitive games match equilibrium game theory predictions

**DOI:** 10.1038/srep05182

**Published:** 2014-06-05

**Authors:** Christopher Flynn Martin, Rahul Bhui, Peter Bossaerts, Tetsuro Matsuzawa, Colin Camerer

**Affiliations:** 1 Department of Brain and Behavioral Sciences, Kyoto University Primate Research Institute, Inuyama, Aichi 484-8506, Japan; 2 Division of the Humanities and Social Sciences, Caltech, Pasadena CA 91125, USA; 3 Department of Finance, University of Utah, Salt Lake City, UT 84112 USA; 4 Computation & Neural Systems, Caltech

## Abstract

The capacity for strategic thinking about the payoff-relevant actions of conspecifics is not well understood across species. We use game theory to make predictions about choices and temporal dynamics in three abstract competitive situations with chimpanzee participants. Frequencies of chimpanzee choices are extremely close to equilibrium (accurate-guessing) predictions, and shift as payoffs change, just as equilibrium theory predicts. The chimpanzee choices are also closer to the equilibrium prediction, and more responsive to past history and payoff changes, than two samples of human choices from experiments in which humans were also initially uninformed about opponent payoffs and could not communicate verbally. The results are consistent with a tentative interpretation of game theory as explaining evolved behavior, with the additional hypothesis that chimpanzees may retain or practice a specialized capacity to adjust strategy choice during competition to perform at least as well as, or better than, humans have.

Interaction among organisms, in which each organism's action influences the fitness reward of the other, is ubiquitous in biological life. However, the ability of different species to think about payoff-relevant actions of conspecifics and respond to action histories is not well understood. In this study, game theory is used to make predictions about choices and temporal dynamics in three abstract competitive situations with chimpanzee and human participants. We show that frequencies of chimpanzee choices are close to equilibrium game theory predictions, and shift as payoffs change, just as equilibrium (accurate-guessing) theory predicts. This is a surprising result. Equilibrium predictions assume mathematically that all organisms making choices correctly anticipate what others do. Dozens of studies with human subjects show substantial deviations from that idealized equilibrium state. However, chimpanzee choices in our data are also closer to the equilibrium prediction, and are more responsive to past history and to payoff changes, than human choices. These results show how game theory can be consistent with evolved behavior^1^, assuming that chimpanzees have a specialized capacity to adjust strategy choice during competition, which appears to be practiced in ontogenetic development. This capacity makes their choices at least as strategic as human choices^2^,^3^,^4^.

We see how our closest genetic relatives, chimpanzees (*Pan troglodytes*), behave as they make choices in incentivized competitive strategic interactions in within-species competition. The interactions are referred to as “games”. Game theory is used to make predictions about possible behavioral outcomes. A chimpanzee-human comparison in repeated competitive games is inspired by the “cognitive tradeoff hypothesis”. This hypothesis is that cortical growth and specialization for distinctly human cognitive capacities (such as language and categorization) conceivably reduced more basic capacities, such as detailed perception and pattern recognition, useful for tracking opponent choice during competition^3^. Those displaced capacities may be better preserved, and more practiced during species development, in species for which those capacities are especially valuable (or “protean”^2^). Intra-group competition is clearly important in chimpanzee societies for establishing a dominance hierarchy^4^, whereas large-scale cooperation is a human specialty. According to the cognitive tradeoff hypothesis, game theory models may better describe chimpanzee behavior than human behavior.

Games are mathematical distillations of the basic action-payoff structures of ecologically valid situations. Interactive experimental strategic games with payoffs have been used with primates and apes to assess prosociality and coordination of mutually beneficial actions^5^,^6^,^7^ and have been used in hundreds of human studies^8^.

We use a common protocol for both animals and humans (1; Methods), in which players were not initially informed of their opponents' payoffs. The games are direct and competitive: Joint actions create one winner and one loser. Both players can either press Left or Right touch-panel buttons (Fig. 1). There are two roles: A Matcher player earns a payoff if their choices match (e.g., Left-Left). A Mismatcher earns a payoff if their choices mismatch (e.g., Right-Left). Joint actions of both players immediately determine their payoff through the shared touch-panel software (there is no subject-experimenter interaction; cf.^5^). Ours is the first experiment in which chimpanzees compete directly with other conspecifics for competitively-determined payoffs, and their behavior is compared to human-human interaction.

Game theory offers a benchmark of optimal performance: Players acting individually earn the most if they guess accurately what others do, and if they choose strategies with maximal expected payoff given those guesses. If both players do so, their choices form a “Nash equilibrium” (NE), a pattern of play in which choices optimally anticipate what others are likely to do.

## Results

The behavioral results answer two questions: (1) How close are the frequencies of Left and Right choices, P(L) and P(R), to the game theory NE predictions?; and (2) How predictably do current choices respond to changes in the opponent's history and to payoff changes?

**Figure 2:**
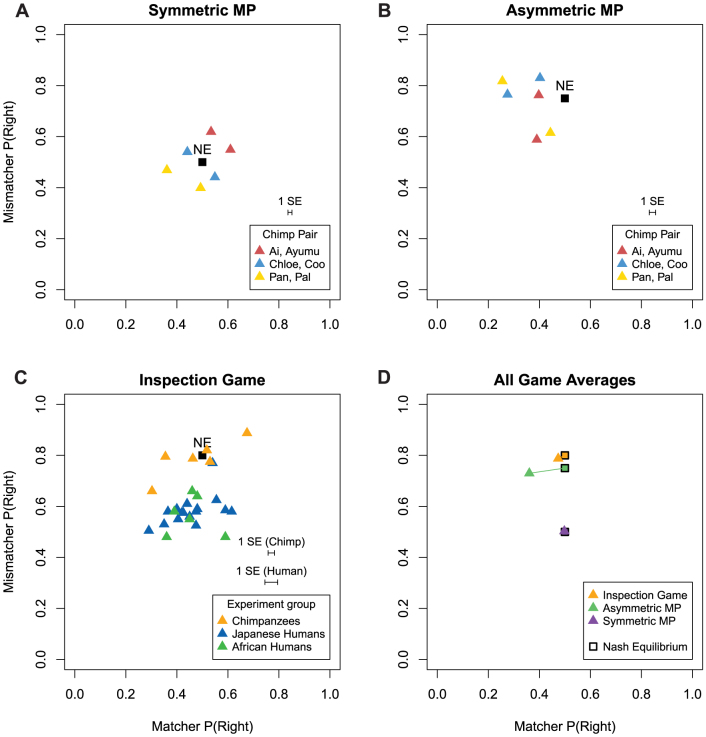
Frequencies of R choices for all pairs in both roles show that chimpanzee behavior is close to game theoretic (NE) predictions. (A, B) Chimpanzees in the symmetric and asymmetric MP games. (C) Chimpanzees and two human groups in the Inspection game. Deviations from Nash equilibrium among chimpanzees average .02 (individual std error .025); deviations among humans average .13 (individual std error .059). Two-sample t-test for the difference in absolute deviation is t(23) = 6.38 (p < .001). (D) Average behavior over all chimpanzees compared to NE for all three games.

Figs. 2a–c plot the overall frequencies of R choice, P(R), by individual chimpanzee subjects in the three games, along with the Nash equilibrium (NE) prediction. The theory predicts that Matchers will choose the Right (R) box on the touch-panel half the time, P(R) = .50, in all three games. The theory also predicts that Mismatchers will vary the frequency of choosing the Right box, P(R), across the three games (see Supplementary Information). These NE predictions are *extremely* counterintuitive: In this class of games, the payoffs of Matcher subjects change. However, their predicted behavior should *not* change across the games (they are predicted to choose P(R) = .50 in all three treatments). Instead, the behavior of the Mismatcher subjects *should* change, even though their payoffs do not change.

Fig. 2d plots cross-subject averages from all trials for all three games. This plot shows whether the chimpanzees' behavior changed across the three payoff conditions as predicted by equilibrium theory. The Matcher P(R) rates are generally close to half on average, as equilibrium theory predicts (despite the fact that matcher payoffs change across games). More strikingly, the Mismatchers' P(R) frequencies do shift numerically across games closely in line with theory (though the Mismatchers' payoffs did not change). The overall frequencies of P(R) for Mismatchers are .50, .73, and .79. These frequencies are quite close to the NE predicted frequencies of .50, .75, and .80.

The close match of chimpanzee choice frequencies to game theory predictions is generally closer than in most previous human experiments (Fig. S2). Intrigued by this finding, we conducted additional experiments with two human groups using a protocol to match the chimpanzee and human conditions as closely as we feasibly could (see Methods and Supplementary Information). Fig. 2c plots choice frequencies for two different human groups in the Inspection game 3, which is the only game that chimpanzees and humans both played. Across Matcher and Mismatcher roles, the human average absolute deviation from NE is .135, a number which is comparable to deviations from NE in other competitive human experiments (see Fig. S2). However, the chimpanzees' average deviation is only .020 (cf. Table S1). The chimpanzee choices are very close to the game theory prediction, and are closer than both human groups' choices are. Since the chimpanzees played many more trials than the human groups did, it is important to note that this difference in proximity to the NE prediction also holds when the first 400 trials of the chimpanzee group are matched to the 400 trials the human groups played in the same game (Figs. S5–7).

Why do the chimpanzees so closely approximate game-theoretic equilibrium? One clue is that working memory of experienced chimpanzees is surprisingly good^9^. Chimpanzee choices may converge more rapidly to the game theory prediction because they remember patterns very well, and in some circumstances better than some humans do. We test this hypothesis using statistical analyses to see how strongly choices depend on beliefs based on previous opponent choices (“fictitious play” learning) and on different player roles. In this analysis, choices depends on two behavioral parameters (10, Supplementary Information): One is a “learning rate” (η), which measures sensitivity to recent opponent choices, and influences updates of the expected payoff of the L and R strategies. The second measure is how well choices for each participant can be predicted based on the learning rate and on varying the frequency of R choice in different role conditions (see Methods and Supplementary Information).

**Figure 3:**
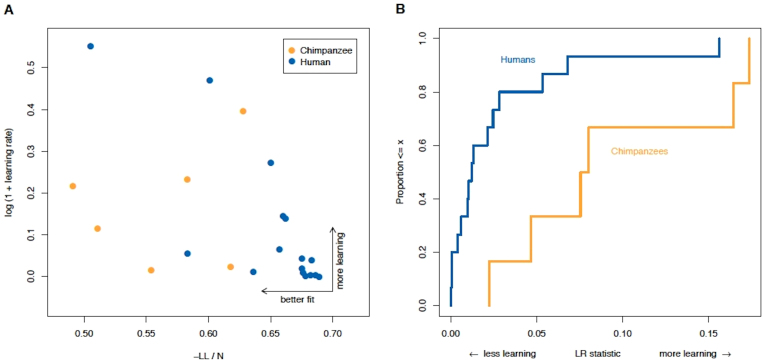
Chimpanzees respond to history and payoff structure more than humans do. (A) History-responsiveness of choices based on learning rate (y-axis) and overall learning model fit which includes Matcher-Mismatcher response differences to payoff structure (x-axis). Chimpanzees have a higher learning rate and a better model fit. (B) Cumulative distribution functions of the likelihood-ratio (LR) statistic (x-axis) showing the improvement in fit of a model with learning compared to a no-learning benchmark. Higher LR numbers indicate more learning; one human outlier removed, see Supplementary Information). The chimpanzee distribution is shifted to the right (i.e., it stochastically dominates the human distribution). This shift means that for every level of detectable learning, more chimpanzees are likely to show that level of learning or greater, compared to humans (p = .040 by an ELR bootstrap test; see Supplementary Information Sect. IV).

Figures 3a–b compare learning rates and predictability across species (excluding, however, one quarter of chimpanzee sessions with extreme bias to one touch-panel side, though aggregate play remains close to NE; see Supplementary Information, Table S4, and Fig. S8). Learning rates and predictability are measured using a dynamic model which accounts for how strongly choices in a trial adjust based on the opponent's previous history and the payoff structure (e.g. Matcher vs Mismatcher role); see Methods and Supplementary Information. The chimpanzees have a higher learning rate (p = .040, nonparametric ELR test; Supplementary Information IV) and much higher predictability than humans (p = .013, t-test; Supplementary Information). These results suggest that the reason the chimpanzees converge more sharply to mutually best-responding (i.e., are closer to NE) is because they adjust to opponent behavior and to changes in incentives more strongly. This comparison does not depend on differences in the numbers of trials the chimpanzee and human groups faced (if anything, learning could be more evident in the shorter trial lengths of the human groups).

Two empirical findings about response times (RTs) are notable.

First, chimpanzee RTs are much faster than human RTs (Fig. S9), about 660 msec versus 900 msec. This may be due to the chimpanzees' extended experience, or their previous experience with touchpad choice in other experiments at PRI. However, it is also consistent with the cognitive tradeoff hypothesis that chimpanzees have retained and developmentally trained skills for memory, computation and response in competitive games.

Second, Matcher RTs are faster than Mismatcher RTs. This difference could arise if Mismatcher choices either involve more complex calculations, or require override of an automatic motor response to match. The motor-override interpretation is consistent with recent evidence in human competitive games, showing that matching an opponent's physical action is automatic—but, remarkably, the difference disappears if both humans wear blindfolds^11^ and persists with more control and incentives^12^.

## Discussion

Our experiments created strategic interactions between pairs of conspecifics using a simple touch-panel protocol. The choice frequencies of food-motivated chimpanzees match game theory predictions as closely as in any species and comparable learning setting ever observed, even when changes in rewards predict highly counterintuitive changes in behavioral choice frequencies. The chimpanzees' choices, compared to the human choices, are closer to those predicted by equilibrium game theory.

There are two broad hypotheses consistent with the facts that chimpanzee choices are much closer to game theory than human choices.

The first hypothesis is that there is a species-based confound in the experimental protocols, and if this confound were eliminated the chimpanzee and human results would then be closer.

There *are* some cross-species confounds in these experiments. We do not think they fully account for direction or magnitude of the chimpanzee-human difference. However, closer matches of human and chimpanzee protocols are conceivable, and would be important to establish more conclusively the differences our data simply suggest.

The chimpanzee subjects are genetically related mother-child pairs and the human pairs are unrelated strangers. But the chimpanzees' kinship should lead to *less* competitive behavior, away from equilibrium; and the difference between chimpanzees and humans does not go in that direction, and in fact goes in the opposite direction (Supplementary Information Sect. VII). The chimpanzees were also motivated by food reward. However, the two human groups were either financially unmotivated (Japan) or very highly motivated (Bossou), and exhibited very similar patterns of play (also comparable to other human groups^1^); so motivation does not explain the behavioral differences.

Furthermore, the deviations from NE among humans observed in this experiment are quite typical of deviations in other human experiments with a variety of information and procedures (including higher monetary stakes) (see 8; Fig. S2). The learning parameter magnitudes for humans also closely match those from an earlier human study with financial motivation using the same Inspection game parameters with full participant knowledge of the game payoffs (10; Fig. 3b; Supplementary Information). Thus, our results from two human groups are not at all unusual compared to previous findings.

The second hypothesis is that chimpanzees actually are as good, or better, at competitive interaction and at adjusting toward equilibrium choices from experience than humans are. Note that this tentative conclusion certainly deserves further investigation since the best evidence of an underlying mechanism — from statistical models of learning — does exclude some unresponsive chimpanzee sessions; then the difference in learning rate between chimpanzees and humans is reasonably significant (p = .040) (although overall predictability is different at p < .001).

Nonetheless, a chimpanzee cognitive match or advantage is plausible because chimpanzees have clear *physical* advantages over humans in strength and speed, which have fitness value in a dominance-mediated social environment. In contrast, human society is relatively egalitarian and non-agonistic, and there is less of a fitness benefit to be gained from strength as a factor in intraspecific social interactions^4^. That chimpanzees are close to NE (in our experiment) and humans are further from NE (here and in other experiments) is consistent with a possible parallel *cognitive* advantage for chimpanzees.

One cognitive advantage emerges in serial ordering tasks requiring memory of briefly exposed spatial displays of Arabic numerals^13^. In one kind of memory masking task, when the lowest numeral of the set is touched, the remaining eight numerals are masked by white squares and subjects are required to touch the masked stimuli in ascending order. One chimpanzee, Ayumu, could at 5.5 years old perform this task above 80% accuracy while touching the first numeral with an average latency of 670 msecs^13^. Humans have not been shown to perform at his level of speed and accuracy on this masking task, though in an easier memory task (the so-called limited-hold task) in which five non-adjacent numerals are briefly exposed for a duration of 210 msecs seconds before being automatically masked, trained humans can achieve similar rates of performance^14^. Matsuzawa^3^ hypothesizes that chimpanzees are better than humans at the masking memory task because human evolution degraded certain memory skills to make room in the brain for development of human language-related skills. The notion that chimpanzees may display some superior cognitive abilities due to a suggested lack of interference from language-related processes is further supported by evidence from comparative eye-tracking studies^15^,^16^. These studies have shown that chimpanzees foveate on the same pictorial elements as humans, but do so in less time by making quicker eye movements. Authors suggest that longer fixation patterns displayed by humans are caused by high-level semantic processing on objects as they are viewed, and that the relative lack of such kinds of language processing in chimpanzees gives them an advantage for making rapid perceptual assessments of visual scenery.

The relatively poor performance of humans, together with the conjectured importance of language for humans, raise issues about the relevance of those game theory experiments in which humans have traditionally been unable to talk to each other. If verbal communication is indeed key to human strategic interaction, it seems that external validity would be enhanced if one lets humans talk. Of course, this challenges classical game theory, which has generally struggled with how to model credibility of verbal communication (“cheap talk”^17^).

Ecological experience and development are likely to play an important role too. In the wild, great apes engage in many competitive strategic interactions such as predatory stalking^18^, young chimpanzee wrestling^19^, border patrolling (which is very much like the Inspection game)^20^, raiding crops from human farms^21^, and play chasing (“tag”^22^). Because competitive payoff games are common in chimpanzee life, evolutionary theory predicts that chimpanzees would have developed cognitive adaptations to detect patterns in opponent behavior and to create predictability in their own behavior. More generally, chimpanzees are capable of strategic thinking in cooperative hunting^23^, sneaky copulation^24^, future planning^25^, and many elements of theory of mind computation^26^. Some have argued that the capacity to randomize effectively evolved because primate predatory behavior and routine social interaction selects for unpredictability in counter-strategies^2^. Experiments also show that chimpanzees are better at competitive tasks than at comparable cooperative ones^27^.

In contrast, humans are relatively highly prosocial and cooperative. As human children begin to speak and acculturate, their play shifts between ages 2 to 4 from solitary and parallel play, to associative and cooperative play^28^. While young chimpanzees continue to hone their competitive skills with constant practice, their young human counterparts shift from competition to verbally-facilitated cooperation.

Our new evidence of more responsive learning and adjustment in competitive experiments by chimpanzees, and evidence of the prevalence of competition in their ecology, supports the interpretation of game theory as an evolutive theory. The evolutive interpretation is that equilibrium game theory will apply particularly well to strategy choices that are finely honed by evolutionary value and (for the chimpanzees) regularly practiced in development and into adulthood. However, it is notable that in our protocol humans are deliberately deprived of an extraordinary cognitive ability -- language. In competitive games language cannot increase group rewards. But in games requiring coordination and cooperation, where language is particularly useful in our ecology, the evolutive prediction is that humans will outperform other species^29^.

## Author Contributions

Design (C.M., C.C., P.B., T.M.); research (C.M.); new analyses (R.B., P.B.); analyzed data (R.B., C.M., P.B., C.C.); wrote paper (C.C., C.M., T.M., R.B.).

## Supplementary Material

Supplementary Information Supplementary Information

## Figures and Tables

**Figure 1:**
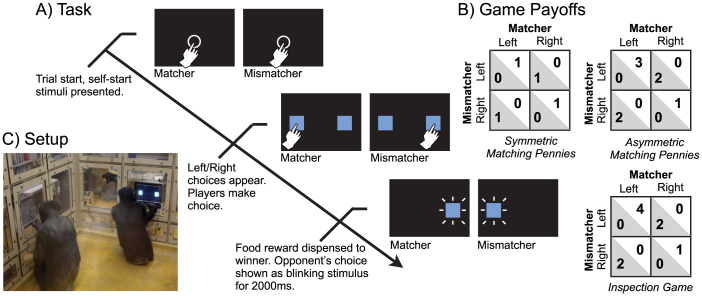
The trial progression, touch-panel setup, and game payoffs. (A) Two players interacting through touch-panel screens are shown a self-start key (circle) at the beginning of each trial. After both players press the start key, two action choices are displayed, represented by squares on the left and right sides of the screens. After both players make a choice, payoffs are dispensed to the winner and both players get feedback about their opponent's choice. (B) Payoff matrices for the 3 games in this study. (C) Subjects sit perpendicular to each other facing touch-panel screens that are embedded in the walls of the experimental booth (photo credit: Chris Martin).
