# Supplementary material for: Chimpanzee choice rates in competitive games match equilibrium game theory predictions
Source: Sci Rep. 2014 Jun 5;4:5182. doi: 10.1038/srep05182 (PMC4046491; doi:10.1038/srep05182)
Supplement: Supplementary Information [file srep05182-s1.pdf]

Supplemental Online Material

Chimpanzee choice rates in competitive games match equilibrium game theory predictions

Christopher Flynn Martin, Rahul Bhui, Peter Bossaerts, Tetsuro Matsuzawa, Colin Camerer

Contents

I. Discussion of Nash equilibrium and boundedly rational theories

II. Previous lab and field evidence from humans

III. Temporal dependence regression

IV. Learning and history-responsiveness

V. An interesting difference between Matcher and Mismatcher response times (RTs)

VI. Histograms of payoffs across subjects and games

VII. A simple model of genetic relatedness does not explain the chimpanzee-human difference

VIII. Further details on Methods

I. Discussion of Nash equilibrium and boundedly rational theories

Nash equilibria in simple games like ours satisfy two properties: (a) Players accurately guess what strategies (or mixtures of strategies) others will play; and (b) players choose the optimal strategy with the highest expected payoff—their "best response"—given their (accurate) beliefs from (a).

In competitive games like those we study, the Nash equilibrium mixtures are proportions which lead to (weak) mutual best responses. For the Inspection game, denote NE Matcher P(Left) by p* and Mismatcher P(Left) by q*. The values of p* and q* will satisfy 2p*=2(1-p*) and 4q*=1(1-q*), or p*=.5, q*=.2.

Note that the mixture probabilities for one player depend **only** on the payoffs of the other player. For example, if the (Left, Left) Matcher payoff was X (rather than 4), the NE mixtures would be p*=.5 (i.e., the Matcher is not predicted to change

behavior at all) and $q^*=1/(X+1)$ (i.e., the Mismatcher is predicted to choose Left less often, as if to deny the Matcher the high X payoff). This is a counter-intuitive feature. Any learning algorithm that is guided by received payoffs (such as reinforcement learning) will therefore adapt, at least in the short-run, in the wrong direction.

Besides learning theories (1), two prominent types of boundedly rational theories have been explored since the mid-1990s (and see (2) for newer theories; but cf. (3)).

"Quantal response equilibrium" (QRE) retains assumption (a) but relaxes the optimization condition (b) to allow "softmax" stochastic imperfections in perceiving and responding to payoff differences (4). This can be seen as a biologically plausible hybrid that combines the formal precision of assumption (a) with a reasonable psychophysical constraint on the ability to produce a perfectly optimizing response. QRE typically uses a single parameter (λ) to encode sensitivity of responses; when the parameter is at its maximal value then QRE is equivalent to NE.

Another class of "cognitive hierarchy" (CH) (or level-k) theories accounts for limited strategic thinking by maintaining the optimality condition (b) and relaxing the assumption of accurate beliefs (a) (5-8). Simple level-0 subjects choose using an intuitive heuristic with no cognition about likely choices of others. Higher level subjects guess accurately what lower-level subjects are likely to do and optimize. More levels of strategic thinking generally correspond to a more accurate model of the social environment and higher rewards. In the Camerer, Ho, and Chong (7) variant the frequency of subjects at each level corresponds to a Poisson distribution with mean and variance of τ.

Our paper includes the first test of this wide range of rational and boundedly rationality game theory models using nonhuman behavioral data. Figures S1a-d show the QRE prediction set. It is graphed as a continuous curve spanning values of $\lambda=0$ (random play, P(Left)=.5 for both players) to $\lambda\rightarrow\infty$ (NE). CH predictions are graphed for a single value, $\tau=1.5$ (which fits many experimental and field data sets reasonably well). NE, QRE, and CH all make the same prediction in symmetric matching pennies. For the other two games, the QRE and CH are actually not more accurate than NE for the chimpanzees. However, QRE fits the human Inspection game data more closely.

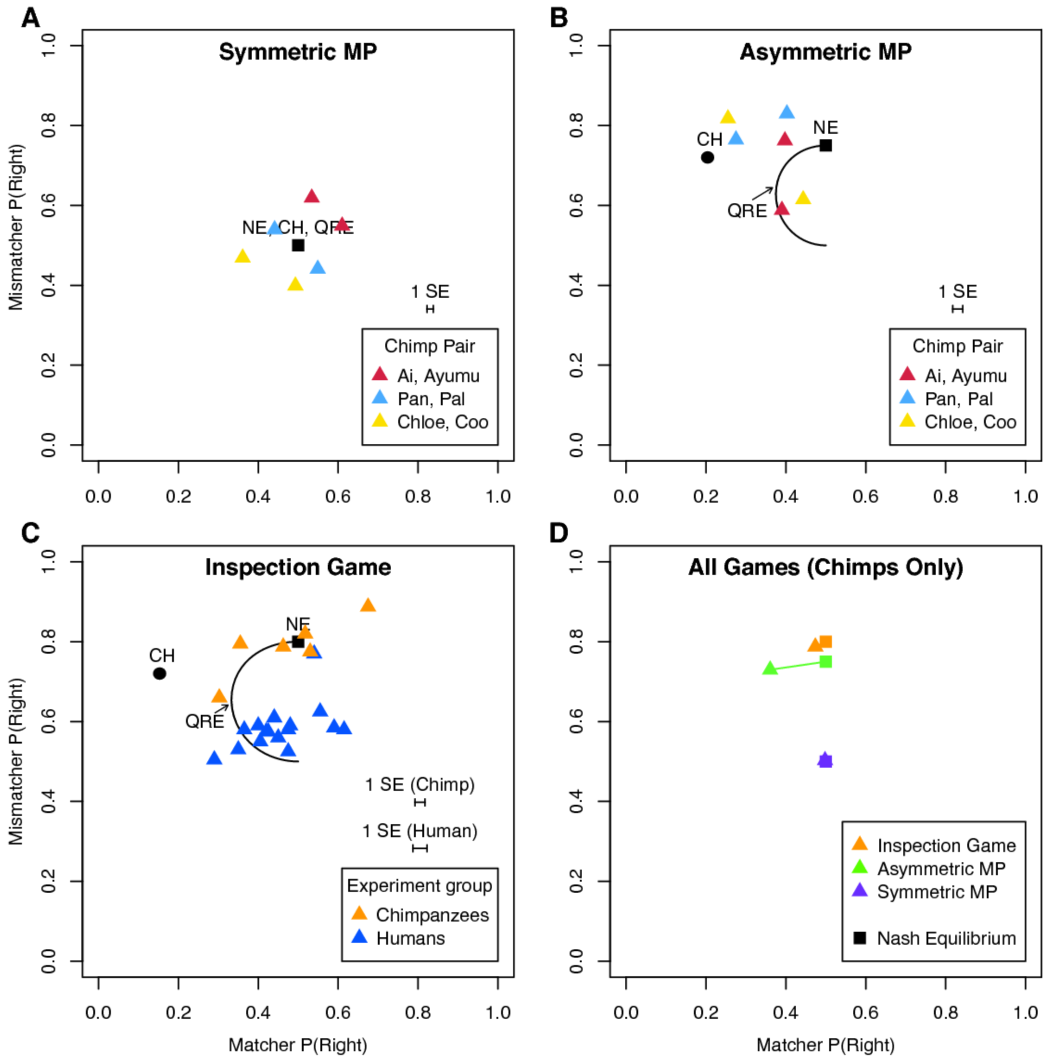

Fig. S1: Frequencies of R choices for all pairs in both roles compared to NE, QRE, and CH predictions. CH predictions are for τ=1.5, while QRE predictions trace out a curve based on the range of values of λ from 0 to ∞.

These results are surprising because QRE and CH typically reliably fit human data as accurately as NE (correcting for their extra degree of freedom, of course). The fact that the chimpanzees are so close to NE in general, and their behavior is *not* well described by QRE and CH, also supports our conclusion that the experienced chimpanzees seem to have some ability to choose NE mixtures which is apparently superior to that of humans, at least in these simple games.

II. Previous lab and field evidence from humans

Many studies with human subjects have examined how well behavior corresponds to NE predictions. This section is abridged from a longer discussion in Camerer (1)

(chapter 3). The empirical background is important for establishing that, for humans, there are typically substantial deviations between NE predicted frequencies and human choices, and that choices are typically not independent over time either.

The earliest studies were conducted in the 1950s, shortly after many important ideas were consolidated and extended in Von Neumann and Morgenstern's (1944) landmark book. John Nash himself conducted some informal experiments during a famous summer at the RAND Corporation in Santa Monica, California. Nash was reportedly discouraged that subjects playing games repeatedly did not show behavior predicted by theory: "The experiment, which was conducted over a two-day period, was designed to test how well different theories of coalitions and bargaining held up when real people were making the decisions. … For the designers of the experiment … the results merely cast doubt on the predictive power of game theory and undermined whatever confidence they still had in the subject."(9)

In the 1960s similar early experimental results were discouraging. However, subjects were often not financially motivated and sometimes played computerized opponents. One striking result (10) showed that people were capable of mixing game-theoretically in a special setting: In their experiment subjects chose first, picked an explicit distribution of strategies (a truly mixed strategy), then the computer observed their mixture and selected a best response. The only way for subjects to win is to choose the equilibrium mixture (since any other choice will be instantly exploited by the computer). In this special setting, they were able to hone in very precisely on NE mixing (65% were playing the exact mixture by the end of a five-game sequence).

These discouraging results turned attention away from mixed-strategy games. Game theorists began to actively research games with private information, and repeated games. A revival of interest in competitive games began with O'Neill's (11) elegant design, a 4x4 game played 500 periods. He reported overall frequencies of play that were much closer to those predicted by NE than the early c. 1960s studies.

However, O'Neill's data were reanalyzed by Brown and Rosenthal (12). They used more careful tests to show that choices often depend strongly on previous choices and previous outcomes (i.e., independence is violated). (The tests they used are the same ones we conducted, reported below in this Supplemental material section III). Others closely replicated these results in games similar in structure.

While there are clearly reliable deviations between NE and human choice, it is notable that the deviations are often small in magnitude, and across different strategies and games there is a substantial correlation between NE predictions and actual choice frequencies. Intuitively, if one strategy X is predicted to be chosen more often than another strategy Y, then X is almost always chosen more often. A glimpse of several studies illustrating the accuracy of this theory-behavior

correspondence comes from a figure in Camerer (1), reprinted with our human data added as Fig. S2 below.

The correlation between predictions and behavior is .84. The mean absolute deviation between predictions and data is .067. Furthermore, keep in mind that predictions usually depend on auxiliary assumptions like neutrality toward risk; if those assumptions are violated then the behavior *should* be a little different than predicted. These results are therefore quite positive in establishing some predictive value of Nash equilibrium predictions. A notable set of experiments with a similarly positive conclusion is Binmore et al. (13). One lesson from these data, then, is that under some experimental conditions behavior close to Nash equilibrium choice can occur.

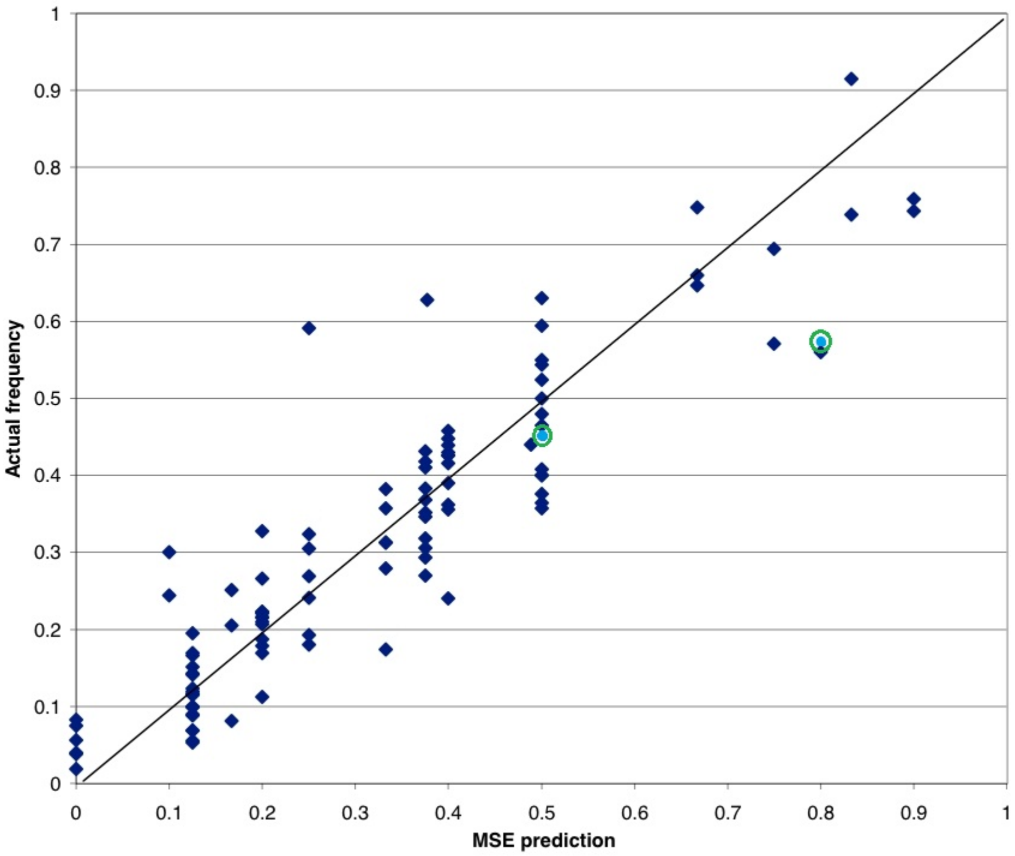

Fig. S2: A cross-study comparison of actual strategy choices frequencies with predictions from Nash mixed-strategy equilibrium (MSE). Each data point is one strategy from one study. The light blue circles represent human data reported in this study. See Camerer (1, chapter 3) for details.

The next important wave of research sought to test whether typical findings in highly-controlled lab settings were also evident in naturally-occurring settings

where randomization is expected. (The quality of field-lab correspondence is often of interest, since economists hope to discover theories which work equally well in highly-controlled artificial lab settings and in field settings with similar features. Camerer (14) discusses the ideas and debate about this topic within experimental economics (see also Heckman and Falk (15)). He also surveys the best available studies. Those studies generally show good correspondence between patterns in field data and patterns in closely-matched lab settings.) Most of the studies use zero-sum competitive sporting events, in which repeatedly playing the same strategy predictably—such as always serving to the same side of the service box in tennis—would typically be noticed and exploited by an opponent.

Early studies of tennis (16-17) and soccer (18-22) found that players' frequencies corresponded fairly closely to those predicted by a NE analysis, and that choices were also roughly independent. The Palacios-Huerta and Volij study is particularly impressive because they are able to match data from actual play on the field from one group of players (in European teams) with laboratory behavior of some players from that group (although not matching the same players' field and lab data). Importantly, PHV also found that high school students as a group behaved less game theoretically than the soccer pros, except that high school students with substantial experience playing soccer were much closer to game-theoretic. However, a reanalysis by Wooders (1) later showed a higher degree of statistical dependence than shown by PHV.

Levitt, List, and Reiley (24) compared behavior of poker, bridge, and soccer players (from US teams) in abstract games conducted off the field. They find substantial deviation from NE and violations of independence. However, PHV noted (personal communication) that the soccer players playing for US teams in the sample were less likely than their counterparts in PHV to actually randomize independently in the field. (The key point here is that the best players, and perhaps the best randomizers, play in soccer-crazy Europe rather than in the US.)

Another field study used a simple lottery played in Sweden by about 50,000 people per day, over seven weeks (25). Participants in the "LUPI" lottery paid 1 euro to pick an integer from 1 to 99,999. The lowest unique positive integer won 10,000 euros. The symmetric NE has a dramatic shape, with numbers from 1 to 5513 being chosen almost equally often, but with slightly declining probability (i.e., 1 is most probable, 2 is slightly less probable, etc.). A bold prediction is that numbers above 5000—a range that includes 95% of all available numbers—should rarely be chosen. The actual behavior is not far from the NE prediction and converges over the seven weeks toward the statistical prediction of the NE prediction (e.g., the mean, variance, and other statistics all move toward NE). In a scaled-down laboratory replication behavior is even closer to NE, even before there is much feedback to learn from.

The general picture from these decades of field and lab studies is that people are capable of approximating Nash mixtures (and certainly of moving in their direction with learning), but that substantial deviations are to be expected. For simple matrix games like those we study, an absolute deviation of 0.05-0.10 between NE

prediction and actual frequency is to be expected for human subjects. The average absolute deviations in the Inspection Game 3 are 0.05 and 0.22, which are comparable to these guesses from many other studies. For chimpanzees, the average across all roles and games is 0.033 (compared to 0.135 for humans).

Table S1: Absolute deviations between NE predictions and average overall frequencies by role, game and species

Deviation (to 2 digits)	**Matcher**	**Mismatcher**
**Sym Chimps**	0	0
**Asym Chimps**	0.14	0.02
**Insp Chimps**	0.03	0.01
**Insp Humans**	0.05	0.22

III. Temporal dependence regression

The histograms (Figs. S3, S4) show the results of a simple test comparing the number of switches in each subject's time series of L-R responses to the number of expected assuming statistical independence. The switching rate histograms for the game-role pairs from the symmetric and asymmetric matching pennies payoff games are shown in Fig. S3 below. They show a little more deviation from random independent play.

Individual 95% confidence intervals for each subject-session uses the mixture probabilities for that subject-session, which imply the mean and variance of the number of runs under the hypothesis of independence (the basis for a Wald-Wolfowitz runs test: Let the number of L choices = n, R choices = m. Then the mean = 2nm/(n+m) + 1 and variance = $2nm(2nm - n - m)/((n + m)^2 (n + m - 1))$) . The number of runs is asymptotically normal, providing a 95% confidence interval for each subject-session with that mean and variance. These 95% confidence intervals were averaged to produce the confidence intervals shown in Figs. S3 and S4.

Our version of the Brown-Rosenthal (BR) equation is

$$R_{t+1} = a_0 + a_1 R_t + a_2 R_{t-1} + b_0 R^*_{t+1} + b_1 R^*_t + b_2 R^*_{t-1} + c_1 W_t + c_2 W_{t-1}$$

where R_t is the player's choice, R^*_t is the opponent's choice, and W_t denotes the winner in period *t*. This logit regression tests for a variety of temporal dependence effects.

Table S2 shows the percentage of role-subject session time series which yield BR coefficients that are significant at the 5% level, for each group of coefficients. For example, for human matchers (role m), 50% of the 16 subjects' regressions

indicated significant dependence of a player's choices on the previous two opponent choices. A joint test for all effects of previous outcomes and choices (the bottom row of the Table) indicates that in almost all cases some of the coefficients are significantly nonzero, when tested together jointly.

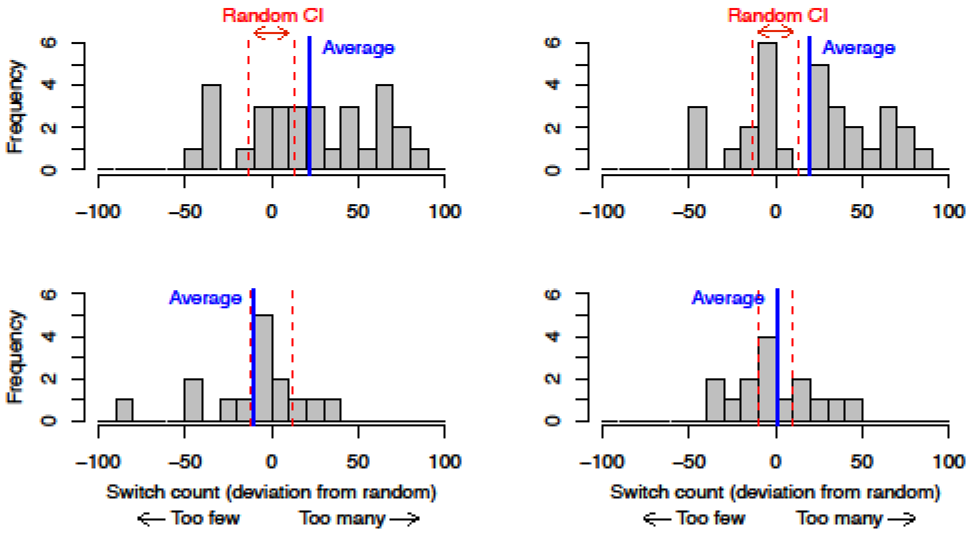

Fig. S3: Switch deviations for chimpanzees in symmetric matching pennies (top row) and asymmetric matching pennies (bottom row). Matchers (left) and Mismatchers (right) are plotted separately.

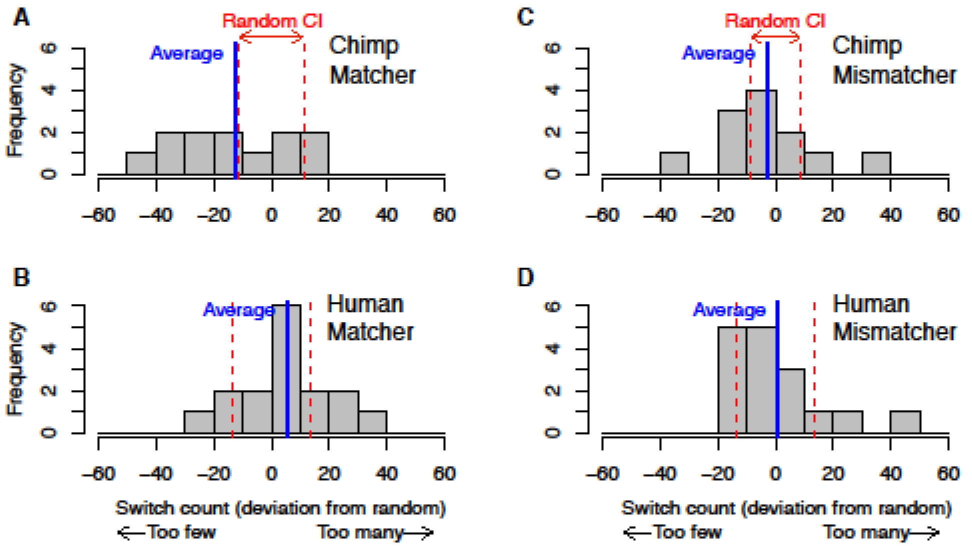

Fig. S4: Switch deviations for chimpanzees (top row) and humans (bottom row) in the inspection game. Matchers (left) and Mismatchers (right) are plotted separately.

Importantly, however, the human and chimp percentage differences in significant dependence rates (shown in the right-hand columns) are generally close together. Z-tests of the difference in percentages across chimps and humans do not indicate any strong differences which persist for both roles.

Table S2: Percentage of significant temporal dependence effects for chimp and human inspection games

		humans		chimps		***Difference (chimps - human)***			
Type of temporal dependence	Coefficient	m	mm	m	mm	m	z-score	mm	z-score
Clairvoyant guessing	b0	0.13	0.13	0.00	0.00	-0.13	-0.91	-0.13	-0.91
Previous opponent choices	b1, b2	0.50	0.63	0.33	0.67	-0.17	-0.70	0.04	0.17
Previous outcomes	c1, c2	0.25	0.25	0.00	0.67	-0.25	-1.35	0.42	2.26
Previous choices and outcomes	b1, b2, c1, c2	0.63	0.63	0.50	0.67	-0.13	-0.53	0.04	0.18
Previous own choices	a1, a2	0.38	0.31	0.50	0.83	0.13	0.53	0.52	2.21
All of the above effects	ai, bi, ci	0.75	0.69	0.83	1.00	0.08	0.42	0.31	1.56
sample size		16	16	6	6				

Note: "m" and "mm" denote matcher and mismatcher roles.

Table S2 shows corresponding percentages (averaging across both Matcher and Mismatcher roles) for the symmetric and asymmetric matching pennies games, and for Brown and Rosenthal’s (12) original analysis of human data (playing 500 trials). Both human data results, and the chimp inspection game, are comparable in the rates of significant dependence.

Table S3: Percentage of significant temporal dependence effects for all chimp and human games, and original Brown-Rosenthal (1990) human data

Type of temporal dependence	Coefficient	Symmetric Chimps	Asymmetric Chimps	Inspection Chimps	Inspection Humans	BR (1990) Humans
Clairvoyant guessing	b0	0.17	0.33	0.00	0.13	0.08
Previous opponent choices	b1, b2	0.83	0.83	0.50	0.56	0.30
Previous outcomes	c1, c2	0.67	0.33	0.33	0.25	0.42
Previous choices and outcomes	b1, b2, c1, (	0.83	0.92	0.58	0.63	0.44
Previous own choices	a1, a2	1.00	0.83	0.67	0.34	0.48
All effects	ai, bi, ci	1.00	1.00	0.92	0.72	0.62
sample size		12	12	12	32	

IV. Learning and history-responsiveness

In this section we present binned data showing the entire time series of behavior (averaged across subjects within each species) as evidence about learning.

First we simply present frequencies P(R) in blocks of 10 trials, averaged across all participants within each species, in Figs. S5-6. There is clearly some movement

toward the Nash equilibrium (denoted by the solid line marked NE). Sometimes there is overshooting. Recall that the three games for chimpanzees were presented in order (not counterbalanced, to avoid confusion) as described in the text materials. Therefore, the Inspection game came last for chimpanzees and first (and only) for humans. To control for experience it is therefore useful to examine P(R) for the mismatchers (who play unequal frequencies in NE) in the earliest learning trials.

Figure S6 indicates just a modest amount of human learning across trials. A linear regression has a slope over 40 blocks of 0.0015 ($p = .051$). The corresponding learning rate for the first chimpanzee game with unequal NE predictions (the asymmetric MP game) is six times higher, 0.0089 ($p < 10e\text{-}9$),

Together, these comparisons show that looking at the first 400 trials played by humans in the Inspection game, and by chimpanzees in the Inspection game or Asymmetric matching pennies, it is not the case that the humans learn quickly in 400 trials but cannot catch up to the longer stretch of trials available to the chimpanzees. The chimpanzees learn faster in the first 400 trials of each game they play.

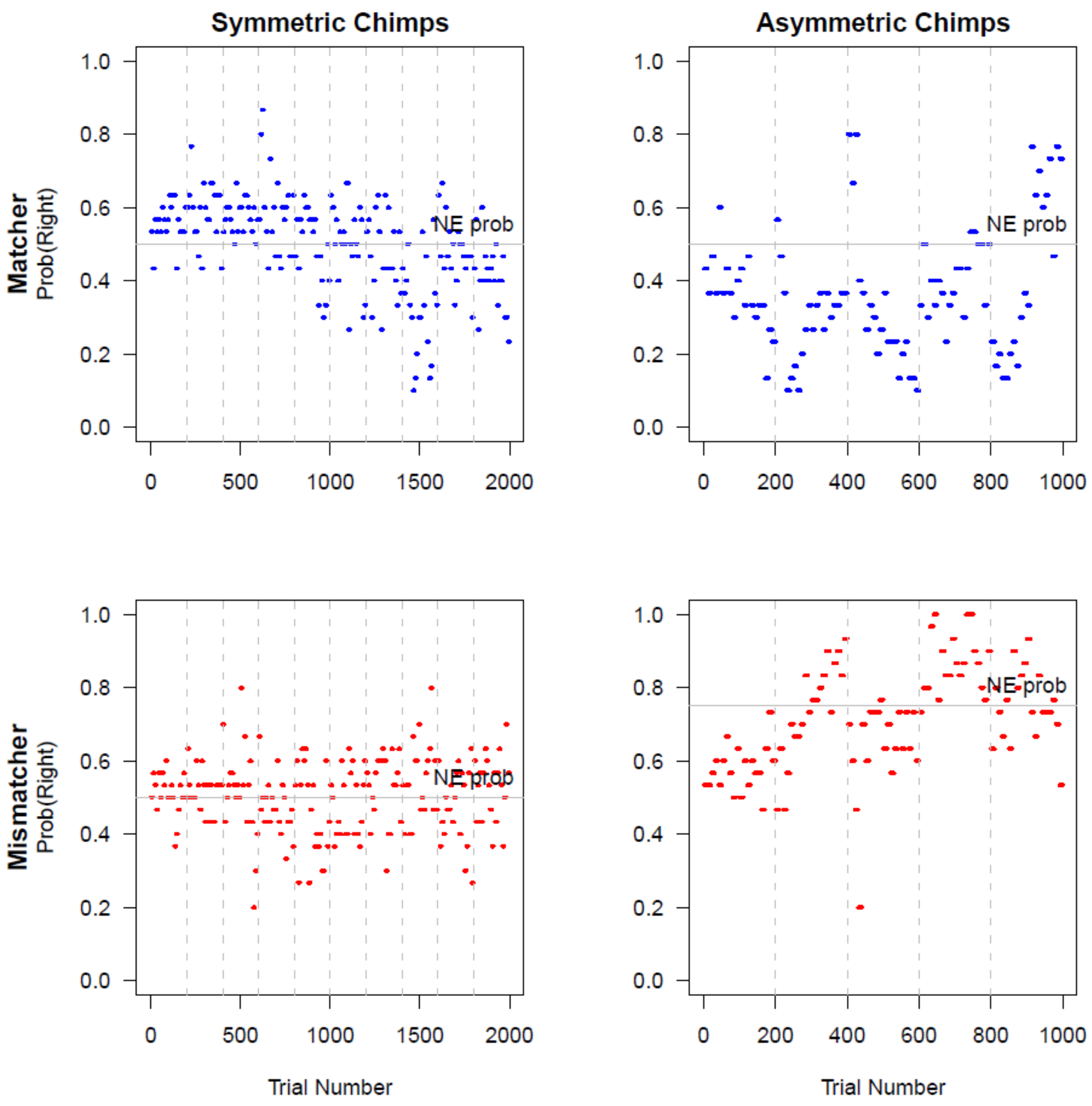

Fig. S5: Population averages of P(Right) across all sessions, in 10-trial blocks (NE marked with a solid line). In the asymmetric game, Mismatcher chimpanzees adjust close to NE after 300 trials.

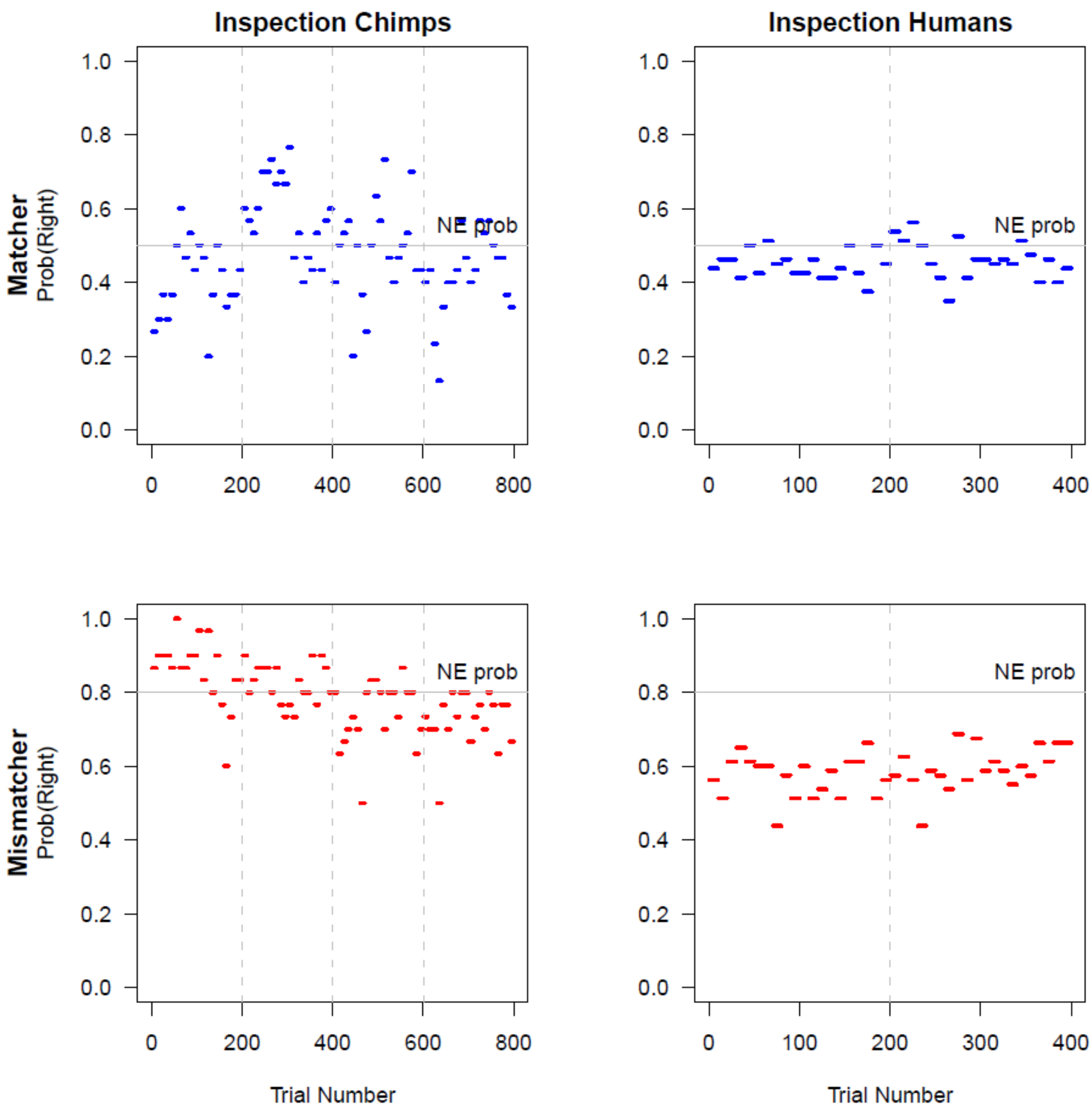

Fig. S6: Population averages of P(Right) across all sessions of Inspection game for both chimpanzees (Left) and humans (Right), in 10-trial blocks (NE marked with a solid line). Mismatcher chimpanzees are already close to NE mixing probabilities in early trials (probably due to spillover from similar play in the preceding Asymmetric MP game). However, Mismatcher human subjects do not adjust much across 400 trials (compared to chimpanzee adjustment in the Asymmetric MP game, which was the first game they faced with unequal NE mixtures).

What is crucial for present purposes is what the overall chimpanzee frequencies look like for the first 400 trials of each game they play. This comparison is different than in Fig. 2d, because it matches the human experience with the comparable first 400 trials of chimpanzee behavior. Figure S7 shows the analogous graph using only 400 trials. The 400-trial Inspection game results are almost identical to those using all the trials, and are still close to NE and far from the human data. Thus, the conclusion of the paper that chimpanzees behave more game-theoretically than humans (in the sense of being closer to NE) still holds.

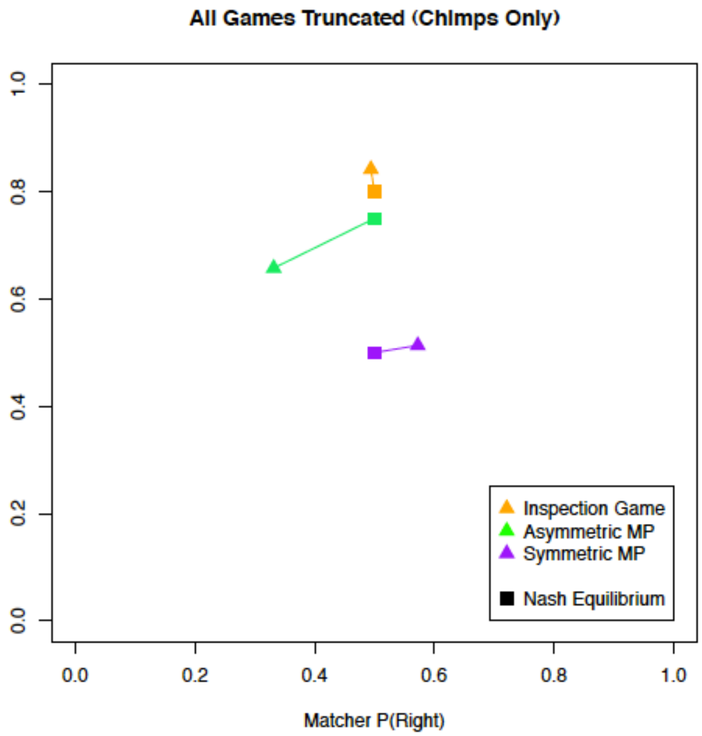

Fig. S7: Analogous reporting to text Fig. 2d using only the first 400 trials of all games.

Figures S5-6 show that variation occurs across time, but does not indicate how learning occurs. A core prediction of the cognitive tradeoff or social protean view is that the chimpanzees may actually be superior to humans in tracking short-term histories and responding to them. From this perspective, the temporal dependence of choices shown in Tables S2-S3) is not necessarily unstrategic, since subjects who are learning by payoff reinforcement or belief updating **should** make choices that depend on histories. To see whether the participants' choices depend on learning studied commonly in human game theory, we use a method developed by Hampton et al. (26).

Fictitious play and estimation

Under (first order) fictitious play, an agent presumes his opponent is playing a mixed strategy where the mixing probability for a given action is inferred from the

history of the opponent's plays. These beliefs are updated based on prediction error – in this case, the difference between the actual action taken and the belief about the probability of that action. The agent then best-responds – chooses the strategy with highest expected payoff – to that opposing mixed strategy. (Our use of the logistic choice rule implies that the agent "better-responds" by playing better actions more often and not always taking the absolute best choice.)

Choice rule: Supposing there are two possible actions, a and b, the probability of choosing action a is given by the logistic function operating on the difference in anticipated values:

$$p^a = f(V^a - V^b) = \frac{1}{1 + e^{-(V^a - V^b)+\alpha}}.$$

In this model, the anticipated value of an action is equal to the expected value of the action given the opponent's mixing probability:

$$V^a = q^*\pi(a,a) + (1 - q^*)\pi(a,b)$$

where q^* is the player's belief (probability estimate) about his opponent's mixing probability for action a (and hence $1 - q^*$ is the player's corresponding belief about b), and $\pi(a,a)$ and $\pi(a,b)$ are the player's payoffs from the (a,a) and (a,b) outcomes. α is a role-dependent lever bias term.

In our matching pennies game, let p be the probability that the Matcher plays Left, and let q be the probability that the Mismatcher plays Left. Further, let p^* be the Mismatcher's belief about the Matcher's probability of playing Left, and let q^* be the Matcher's belief about the Mismatcher's probability of playing Left. The decision of player 1, the Matcher, as a function of his belief about player 2, is

$$p = f(V^L - V^R)^{Matcher}$$

$$= f([\pi(L,L)q^* + \pi(L,R)(1 - q^*)] - [\pi(R,L)q^* + \pi(R,R)(1 - q^*)])$$

and the decision of player 2, the Mismatcher, as a function of her belief about player 1, is

$$q = f(V^L - V^R)^{Mismatcher}$$

$$= f([\pi(L,L)p^* + \pi(R,L)(1 - p^*)] - [\pi(L,R)p^* + \pi(R,R)(1 - p^*)])$$

Updating rule: Calling p_t^* the player's belief about her opponent's probability of playing action a in trial t, the player's belief is updated as follows:

$$p_{t+1}^* = p_t^* + \eta\delta_t^p$$

where the prediction error

$$\delta_t^p = P_t - p_t^*$$

is the difference between an indicator representing whether action a was taken by the opponent in trial t ($P_t = 1$) or not ($P_t = 0$) and the belief p_t^*. Hence

$$p_{t+1}^* = p_t^* + \eta(P_t - p_t^*).$$

We estimate the learning rate parameter η for each agent by fitting the choice probabilities predicted by the model, p_a and p_b, to the actual choices made by the agents, y_a and y_b (indicator variables describing whether or not the corresponding action was taken). The parameters were estimated separately for each pair, for each agent in that pair, by maximizing the log likelihood:

$$\log L = \sum_{games} \left(\sum_{trials} y_a \log p_a + y_b \log p_b \right)^{Chimp\ 1} + \sum_{games} \left(\sum_{trials} y_a \log p_a + y_b \log p_b \right)^{Chimp\ 2}$$

using numerical optimization with starting values from a grid with boundaries (0, 1) for η and (-0.25, 0.25) for α, with increments of 0.25.

Model fits which are consistent with payoff-responsive learning will have substantial values of the learning rate n (above 0.10), typically small lever bias (a < .5 in magnitude) and a substantial mean predicted probability. Although some blocks were excluded in estimation due to excessively perseverative runs, the overall mixture probabilities excluding these blocks remain close to NE, as Fig. S8 shows.

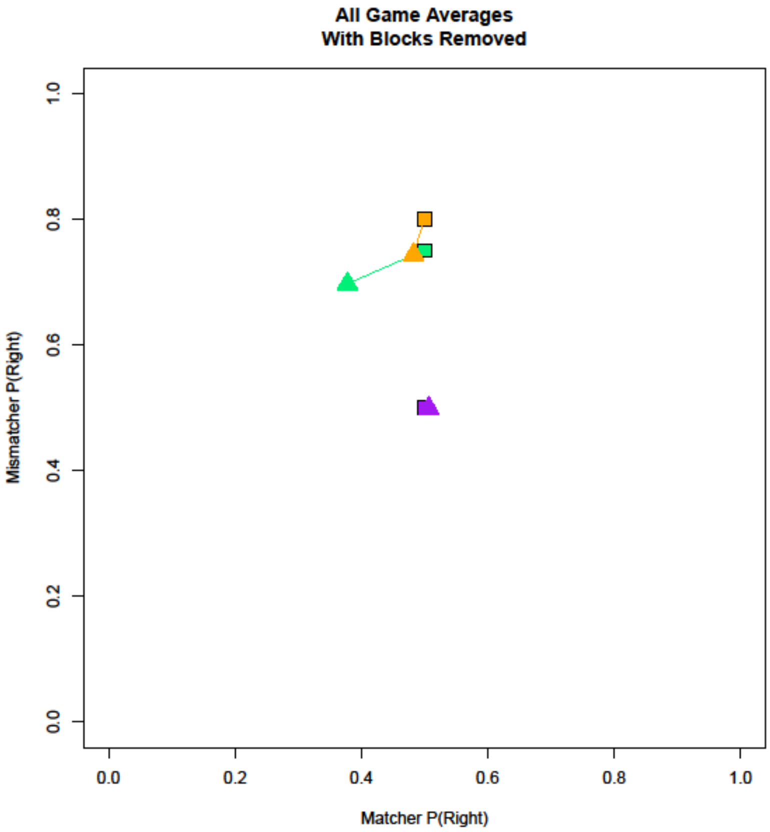

Fig. S8: Analogous reporting to text Fig. 2d using only the blocks included in the learning model estimation.

While the sample sizes are obviously small, there is more evidence of payoff-responsive learning by the chimpanzees. The median learning rate is 0.181 and the median predicted probability is 0.566. For human participants, the median learning rate is only 0.041 and the overall mean predicted probability is 0.512. This difference is not conclusive because of the challenges (described in the main text) of closely matching chimpanzee and human experiments on comprehension and incentives. However, it is consistent with the hypothesis that the chimpanzees are more inclined to using histories of opponents' choices to adjust their own strategy choices (as evidenced by higher learning rates and higher predictability of choices based on opponent history). This difference is consistent with the cognitive tradeoff and social protean hypotheses.

Table S4: Best-fitting learning parameters, Inspection game data only (ordered from low to high learning rate within species).

Pair, player #	Learning rate (η)	Matcher "lever bias" (α^{Mat})	Mismatcher "lever bias" (α^{Mis})	Exp(LL/N)	-LL

Chimpanzees					
1,2	0.014	-0.121	-1.259	0.575	443.1
3,1*	0.022	-0.239	-0.601	0.539	370.6
3,2*	0.121	0.160	-1.842	0.600	306.3
2,1**	0.241	-1.627	-1.545	0.612	196.6
2,2**	0.262	-0.104	-1.524	0.558	233.3
1,1	0.485	0.251	-0.007	0.534	502.4
Median	0.181			0.566	
Humans					
7,2	-0.002	-1.910	-0.226	0.502	275.4
1,2	0.001	-1.690	-0.426	0.508	271.2
2,1	0.002	-1.320	-0.236	0.504	274.3
3,1	0.003	-1.036	-0.084	0.505	272.9
4,1	0.009	-1.525	-0.187	0.509	270.5
6,1	0.011	-0.604	-0.102	0.529	254.4
8,2	0.018	-0.967	-0.633	0.509	269.8
6,2	0.039	-0.920	0.735	0.505	273.0
5,2	0.043	-1.072	-0.085	0.509	269.9
7,1	0.055	-0.721	-1.387	0.558	233.1
4,2	0.066	-0.977	-0.673	0.519	262.7
2,2	0.149	-0.550	-0.239	0.516	264.6
1,1	0.154	-0.657	-0.733	0.517	263.8
3,2	0.313	-1.241	-0.007	0.522	260.1
5,1	0.599	-0.565	-0.394	0.549	240.2
8,1	0.734	-1.903	0.141	0.604	201.9
Median	0.041			0.512	

Note: *denotes 600 observations ** denotes 400 observations (all other chimpanzee players have 800 observation). Some games were excluded due to long perseverative runs of identical choices. The overall chimpanzee analysis uses 3600 out of 4800 available observations. Human data are 400 observations per subject (overall 6400 observations).

Since the humans tend to generate poor model fits, the distribution of estimated learning rates η is skewed and non-Gaussian, so a two-sample t-test assuming Gaussian data is inappropriate.[1] A nonparametric approach compares the two population learning rates by comparing their distributions of likelihood ratio (LR) statistics. The LR statistic (normalized by sample size) calculated for each subject is $-2(LL_r - LL_{ur})$, where the restricted model allows lever bias to be a free parameter, but requires the learning rate to be 0. Comparing the LR then measures how much better-fitting a model with unrestricted learning is. That is, the restricted model just

[1] While the parametric basis for a t-test is unsound, readers might want to know that the resulting p-value for the difference in the human and chimpanzee learning rates has a p-value of .119.

fits the empirical frequencies from players in both roles, but does not allow any dependence on history. The unrestricted model allows both role-dependent frequencies *and* learning. This procedure creates a sample of LR statistics $\{y_t^K\}$ for each group $K \in \{H, C\}$ (human and chimpanzee), from which we remove a human outlier ($p<10^{-15}$, Dixon test). Then we test for the first-order stochastic dominance of chimps over humans. First-order stochastic dominance implies that for any level of improvement in fit from allowing learning, more chimpanzees have an LR statistic higher than that level than humans do. That is, we use the Davidson-Duclos (27) empirical likelihood ratio (ELR) statistic which satisfies

$$\frac{1}{2}LR(z) \\ = N\log N - N_H\log N_H - N_C\log N_C + N_H(z)\log N_H(z) + N_C(z)\log N_C(z) \\ + M_H(z)\log M_H(z) + M_C(z)\log M_C(z) - \big(N_H(z)+N_C(z)\big)\log\big(N_H(z)+N_C(z)\big) \\ - \big(M_H(z)+M_C(z)\big)\log\big(M_H(z)+M_C(z)\big)$$

where N_K is the size of sample K, $N_K(z) = \sum_t \boldsymbol{I}(y_t^K \le z)$ (in the continuous case) is the number of points less than or equal to z in sample K, and $M_K(z) = N_K - N_K(z)$. The ELR statistic is $LR^{1/2}(\tilde{z})$, evaluated at the value of z within the interior of the joint support which minimizes it. Bootstrap samples can be constructed by resampling using probabilities

$$p_t^K = \frac{N_K(\tilde{z}) + N_{-K}(\tilde{z})}{NN_K(\tilde{z})} \text{ for } y_t^K \le \tilde{z} \text{ and } \frac{M_K(\tilde{z}) + M_{-K}(\tilde{z})}{NM_K(\tilde{z})} \text{ for } y_t^K > \tilde{z}$$

where $\tilde{z}$ is the ELR-statistic-minimizing value of z, and denoting the other group by $-K$. For each bootstrap sample, the ELR statistic can be calculated at the minimizing value of z for that sample independently. The ELR statistic from the original data is tested against the distribution consisting of this set of bootstrapped statistics. Doing so yields a p-value of .040. Including the outlier human value yields a p-value of .073, which remains strongly suggestive of a replicable difference (particularly considering the test is conservative, i.e., it has a tendency to under-reject the null hypothesis of nondominance).

The t-test for predictability (exp(LL/N)) works as follows: The numbers in the column (5) for both human and chimp predictability are used as statistics. TTEST in Excel is used, setting optional values to 2-tailed tests for a 2-sample test with unequal variances. The p-value (Welch's t-test) is .013.

V. An interesting difference between Matcher and Mismatcher response times (RTs)

There are some interesting patterns in response times (RTs). Each point in Fig. S9 shows the pair of averaged RTs for each subject, when playing as both Matcher (x-axis) and Mismatcher (y-axis). One evident result is that Mismatcher RTs are nearly *always* longer (i.e., slower) than Matcher reactions. One theory to account for this difference is that, in equilibrium, the Mismatchers have to choose unequal portions

of L and R responses. However, the slight RT difference is even evident in the symmetric games, where L and R responses are predicted to be equally common (and actually are, empirically) for both Matcher and Mismatcher.

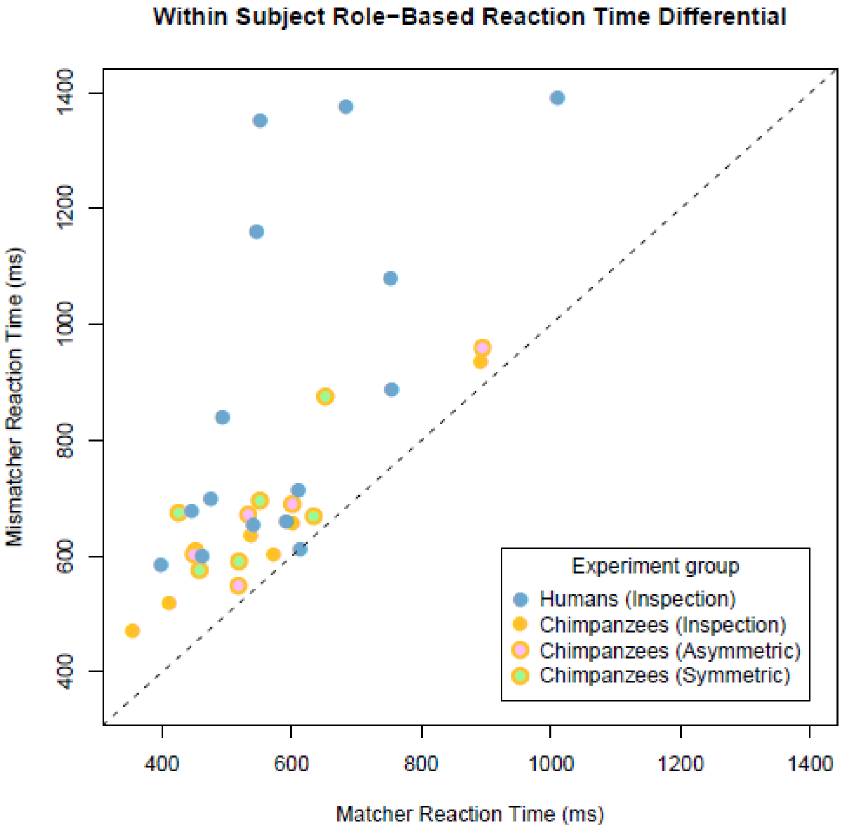

Fig. S9: Average reaction times for individual subjects when playing as Matcher (x-axis) and Mismatcher (y-axis).

We speculate that the RT differential might indicate some kind of highly evolved (and conserved across species) speed for physical imitation of movements, compared to anti-imitation.

Since each subject participated in both roles, as Matcher and Mismatcher, we can compare their RTs in the two different roles. (There is no experience confound, because half of the subjects were in the Matcher role first and half in the Mismatcher role first). A paired sign test for differences in medians for these within-subject RTs rejected the hypothesis of equal medians (median difference 127ms, $p<10^{-8}$) even when including an outlier (Matcher RT 1748ms, Mismatcher RT 1086ms; not shown in Fig. S9).

VI. Histograms of payoffs across subjects and games

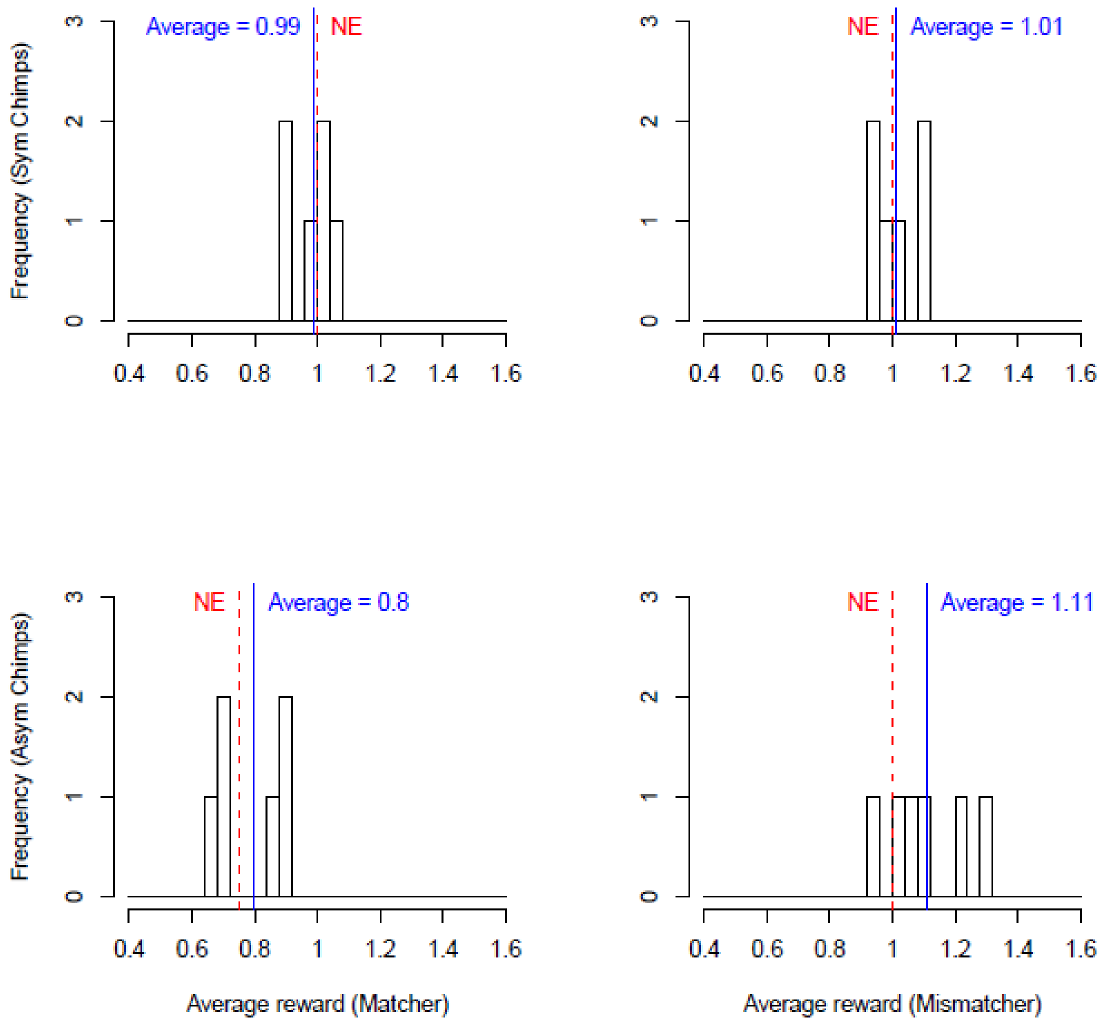

Fig. S10: Average payoff distributions across all chimpanzee-sessions in Matching Pennies and Asymmetric Matching Pennies games.

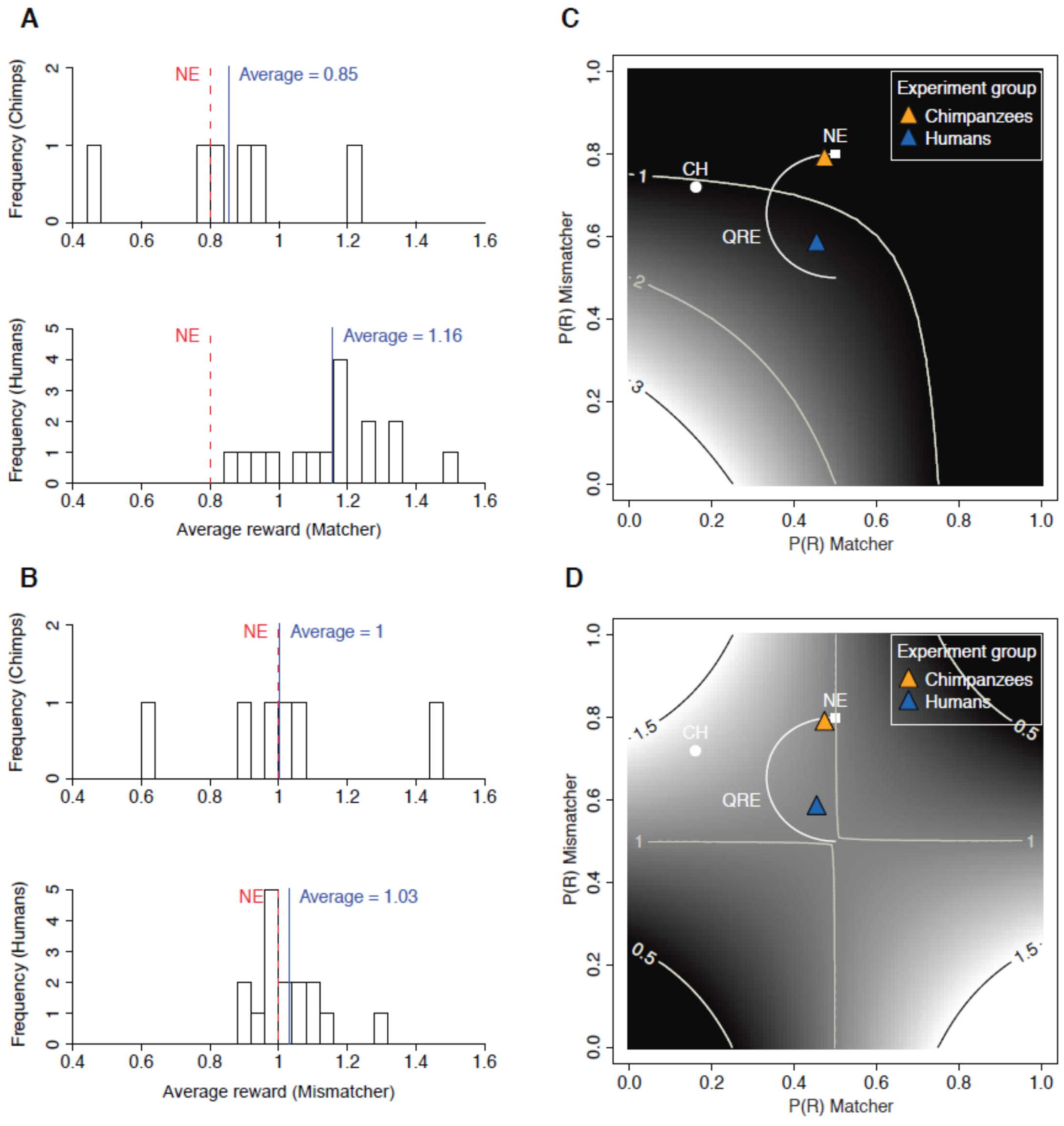

Fig. S11. Average payoff distributions for (A) Matchers and (B) Mismatchers of both species in Inspection games. The only systematic deviation from Nash equilibrium (NE) payoffs is that human Matchers earn more. Grayscale heatmaps (C,D) show payoff contours (higher payoffs are lighter regions). (C) This heatmap for Matcher payoffs shows that human Mismatchers deviate more from NE, which benefits Matchers (i.e. the blue triangle is in a lighter region—a higher payoff-- than the NE). (D) This heatmap for Mismatcher payoffs shows that human Matchers are empirically close (the blue triangle) to the NE prediction of P(R|Matcher)=.5. As a result, there is no substantial difference in payoffs to Mismatchers. Note: Both heatmaps assume that there is no ability coordinate choices between Matchers and Mismatchers in order to maximize joint payoff.

VII. A simple model of genetic relatedness does not explain the chimpanzee-human difference

Because the mother-child chimpanzee pairs are genetically related (and the humans are likely much less related) it is useful to analyze whether the chimpanzee-human differences could be due to departures from self-interest in payoff gain due to relatedness. A natural simple model is to assume that one player earns a fraction r of the other player's payoff, where r is a relatedness coefficient (or, in a model of human altruism, interpreted as sympathy or personal gain from another person's earnings).

Then the payoff matrix looks like this:

		mismatcher	
		L	R
matcher	L	X, rX	2r, 2
	R	2r, 2	Y, rY

Table S5. Matching pennies variants with altruistic spillover of payoffs.

When r=0 it reduces to the purely selfish games. Denote p(R|matcher)=p*(r) and q(R|mismatcher)=q*(R). When the Nash equilibrium probability mixtures are computed for this game, we see:

p*(r)=(2-rX)/(4-r(X+Y)) and q*(r)=(X-2r)/(X+Y-4r)

When X=Y=2 (pure matching pennies Game 1) p*(r)=q*(r)=.5 for all r. In this game, altruism plays no role.

However, for X=3,Y=1 (Game 2, also denoted AMP), changing the value of r traces a continuous line from the selfish (r=0) equilibrium to a point on the boundary. The notation (x,y) denotes (P(R|matcher)=x, P(R|mismatcher)=y. For Game 2, changing the value of r traces an arc from (.5,.75) to the boundary p*(R|matcher)=.25, q*(R|mismatcher)=1.

For X=4, Y=1 (Inspect. Game 3) changing the value of r traces a continuous line from the equilibrium (.5,.80) to the boundary p*(R|matcher)=.0, q*(R|mismatcher)=1.

For both games 2-3, however, something interesting also happens when r>1/2.

In Game 2 (X=3), when r=1/2 choosing L becomes a strictly dominated strategy for the matcher (since Y=1). Then p*(R|matcher)=0 and the mismatcher column player chooses q*(R|mismatcher)=1 for ½<r<2/3. However, when r>2/3 the pair (L,L) becomes dominant for *both* players so there is a jump in the altruism-adjusted Nash equilibrium to (0,0).

In Game 3 (X=4), when r=1/2 the pair (L,L) becomes dominant for both players so there is a jump in the Nash equilibrium to (0,0).

All these effects are shown in Figure S12.

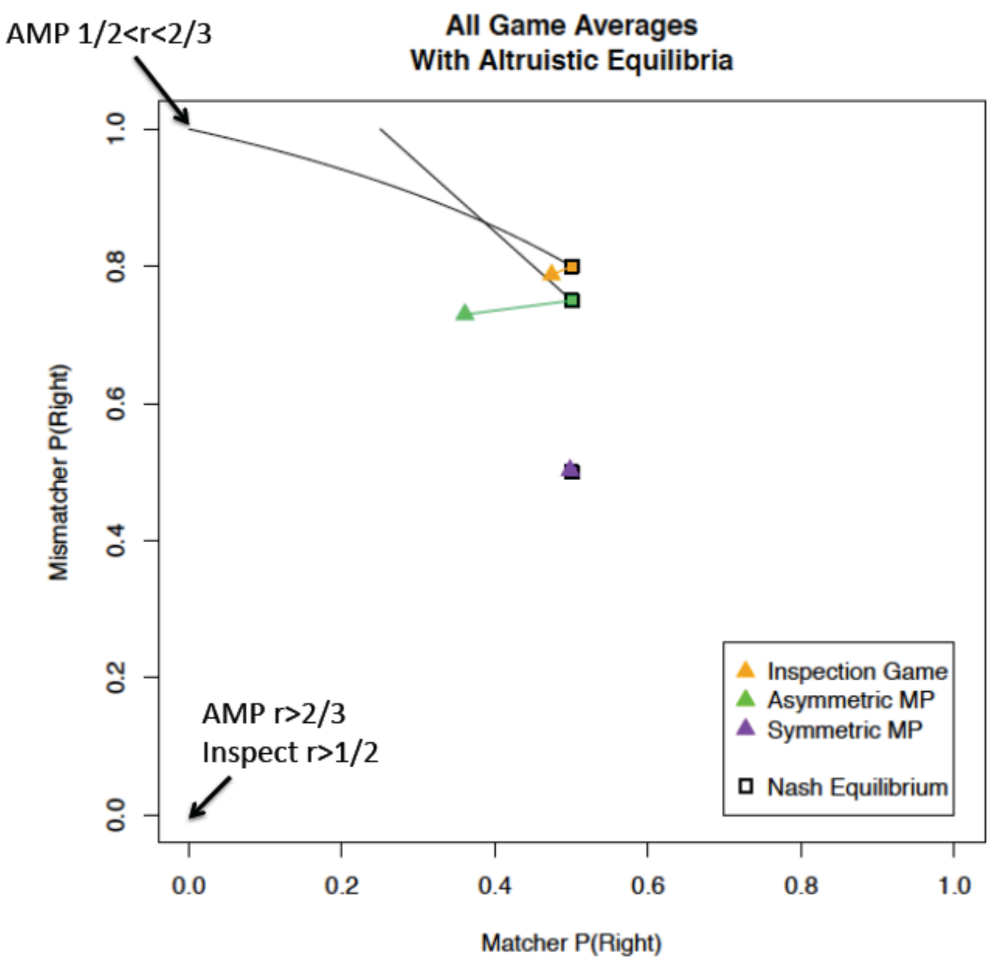

Fig. S12: Nash equilibrium paths (black lines) when utility payoffs are influenced by a parameterized degree of pure altruism. The altruism weight, r, is the weight each player places on the other player's direct reward. Increasing altruism moves equilibrium pairs toward (L, R) (up to a point), hitting a boundary in which q(R|mismatcher)=1. In game 2, when r≥.5 the choices jump to (0,1), then jump again to (0,0) when r≥2/3. In game 3, when r≥.5 the choices jump to (0,0) because the (L,L) is dominant (adjusting for altruism r≥.5). This simple model cannot explain why chimpanzee pairs (who higher genetic relatedness than the human groups), move, across games, toward higher frequencies P(R|mismatcher) with no substantial movement in P(R|matcher). That is, this graph shows why the hypothesis that the chimpanzee-human difference is due to genetic relatedness cannot account for the main difference in choice frequencies.

VIII. Further Details on Methods

Players made choices on pairs of computer touch-panel screens. Each screen displayed two identical stimuli (45mm light blue square buttons) on the left and right sides of the screen (text Figure 1a). If both subjects chose the button on the right, or if both subjects chose the button on the left, then the "Matcher" was rewarded. If the subjects chose buttons on different sides, then the "Mismatcher"

was rewarded. At the end of each trial as the winner was rewarded, a blinking stimulus appeared on the side of each screen that had been pressed by the opponent. No control tests were made to assess the players' comprehension of the meaning of the blinking stimulus as an indication of "other's choice", though use of opponent feedback without comprehension testing is a standard practice for non-human primate decision making tasks (28). The chimpanzee subjects had some visual and much audible feedback about the outcomes of each round, as the winner's feeder made noises to dispense the food. While humans had no visual feedback about the other's payoff, they had audible feedback in the form of the sound of coins dropping into their opponent's food tray. Payoff structures changed across three kinds of games (Figure 1c). Pairs played 200 rounds of a game per session. Chimpanzees switched roles between sessions and played game 1 (symmetric matching pennies) for 10 sessions, game 2 (asymmetric matching pennies) for 5 sessions, and game 3 (inspection game) for 4 sessions. Pairs of humans played game 3 (inspection game) for 2 sessions, switching roles once. The primary aim of the current study was to assess the chimpanzees' behavior in competitive games, with the comparison to humans as a secondary goal. Therefore, the chimpanzees played more games, and more sessions of each game compared to humans. The lack of perfect methodological matching in the number of games and sessions between human and chimpanzee conditions was also due to logistical difficulties, including the possibility of experimental attrition that may have resulted from having humans participate in repeated sessions over the course of many days. During the games, players were seated in an experimental booth facing away from each other (Figure 1b). Universal feeding machines (Biomedica Model BUF-310), delivered 8 by 8mm cubes of apple (or tokens in the case of humans) on a trial-to-trial basis. Humans, but not chimpanzees, had an opaque barrier between them to limit communicative exchanges that likely would have affected their behavior. Since chimpanzees show little or no communication during the games, the barrier was placed for humans in an attempt to replicate the non-communicative context of the chimpanzee condition. A single PC running a Visual Basic 6 program controlled all experimental events involving the two touch-screens and feeders.

Subjects

Six chimpanzees (*Pan Troglodytes*) at the Kyoto University Primate Research Institute voluntarily participated in the experiment. The subjects were three mother-offspring dyads (Ai, a 31 year old female and her 9 year old son Ayumu; Chloe, a 30 year old female and her 9 year old daughter Cleo; and Pan, a 27 year old female and her 9 year old daughter Pal). The mother-offspring dyads were pair-matched with each other for all of the experimental games in this study. All participants had previously taken part in cognitive studies, including social tasks involving food and token sharing (29-30), and a dual touch-panel study in which they observed and copied the behavior of a conspecific model (31). However, the dual touch-panel competitive game in this study was novel to the participants. The 6 participants lived with 7 other chimpanzees in a semi-natural enriched enclosure, and were not food or water deprived during the period of the study. The

chimpanzee study lasted over a period of 30 days, with experimental sessions occurring on average 4 times a week for each subject pair. The use of the chimpanzees during the experimental period adhered to the Guide for the Care and Use of Laboratory Primates (2002) of the Primate Research Institute of Kyoto University.

16 human Japanese participants (13 female) participated in the experiment. The participants were undergraduate and graduate students of Gifu University and Kyoto University. In a player-matched design, pairs of subjects were exposed to 50 training trials in each of the Matcher and Mismatcher roles in order to gain familiarity with the task and payoff structure of the game. Training trials were given to humans, but not to chimpanzees, because the chimpanzees experienced multiple role-reversals between sessions that humans did not, thus allowing the chimpanzees a greater opportunity to identify the payoff structures of each role without explicit training sessions. After the training rounds, humans played 200 rounds in each of the two roles. The experimental design and procedure was identical to that of the chimpanzee task, except that coin tokens were dispensed from the feeders instead of apple pieces, and an opaque barrier was placed between the stations to prevent collusion. In order to maximize parity between chimpanzee and human conditions, the human subjects were not given any verbal instructions prior to the task, and were not told that they were to play a competitive game against each other. After completing the task, participants were given 500-yen (approximately 6 US dollar) gift-cards. The ethical committee of Primate Research Institute of Kyoto University approved the use of human subjects. Methods used with Bossou in Guinea, Africa are described in the main text.

1. Camerer CF (2003) *Behavioral Game Theory: Experiments in Strategic Interaction* (Princeton Univ Press, Princeton, NJ).
2. Selten R, Abbink K, Buchta J, & Sadrieh A (2003) How to play (3 x 3)-games. A strategy method experiment. *Game Econ Behav* 45(1):19-37.
3. Brunner C, Camerer CF, & Goeree JK (2011) Stationary Concepts for Experimental 2 x 2 Games: Comment. *American Economic Review* 101(2):1029-1040.
4. McKelvey RD & Palfrey TR (1995) Quantal Response Equilibria for Normal Form Games. *Game Econ Behav* 10(1):6-38.
5. Nagel R (1995) Unraveling in Guessing Games: An Experimental Study. *The American Economic Review* 85(5):1313-1326.
6. Stahl D, O. & Wilson P, W. (1995) On Players' Models of Other Players: Theory and Experimental Evidence. *Game Econ Behav* 10(1):218-254.
7. Camerer CF, Ho TH, & Chong JK (2004) A cognitive hierarchy model of games. *Quarterly Journal of Economics* 119(3):861-898.
8. Crawford V, Costa-Gomes M, & Iriberri N (2010) Strategic Thinking.
9. Nasar S (1998) *A Beautiful Mind: A Biography of John Forbes Nash Jr* (Simon & Schuster, New York).

10. Kaufman H & Becker GM (1961) Empirical Determination of Game-Theoretical Strategies. *Journal of Experimental Psychology* 61(6):462-&.
11. O'Neill B (1987) Nonmetric Test of the Minimax Theory of Two-Person Zerosum Games. *Proc Natl Acad Sci U S A* 84(7):2106-2109.
12. Brown JN & Rosenthal RW (1990) Testing the Minimax Hypothesis: A Re-Examination of O'Neill's Game Experiment. *Econometrica* 58(5):1065-1081.
13. Binmore, K, Swierzbinsk, J, Proulx, C. (2001). Does Minimax Work? An Experimental Study, *Economic Journal*, 111: 445-464.
14. Camerer C (2012) The Promise and Success of Lab-Field Generalizability in Experimental Economics: A Critical Reply to Levitt and List.
15. Falk A & Heckman JJ (2009) Lab Experiments Are a Major Source of Knowledge in the Social Sciences. *Science* 326(5952):535-538.
16. Walker M & Wooders J (2001) Minimax Play at Wimbledon. *American Economic Review* 91(5):1521-1538.
17. Hsu SH, Huang CY, & Tang CT (2007) Minimax play at Wimbledon: Comment. *American Economic Review* 97(1):517-523.
18. Palacios-Huerta I & Volij O (2008) Experientia Docet: Professionals Play Minimax in Laboratory Experiments. *Econometrica* 76(1):71-115.
19. Chiappori PA, Levitt S, & Groseclose T (2002) Testing Mixed-Strategy Equilibria When Players Are Heterogeneous: The Case of Penalty Kicks in Soccer. *American Economic Review* 92(4):1138-1151.
20. Palacios-Huerta I (2003) Professionals play minimax. *Review of Economic Studies* 70(2):395-415.
21. Moschini GC (2004) Nash equilibrium in strictly competitive games: live play in soccer. *Economics Letters* 85(3):365-371.
22. Azar OH & Bar-Eli M (2011) Do soccer players play the mixed-strategy Nash equilibrium? *Applied Economics* 43(25):3591-3601.
23. Wooders J (2010) Does Experience Teach? Professionals and Minimax Play in the Lab. *Econometrica* 78(3):1143-1154.
24. Levitt SD, List JA, & Reiley DH (2010) What Happens in the Field Stays in the Field: Exploring Whether Professionals Play Minimax in Laboratory Experiments. *Econometrica* 78(4):1413-1434.
25. Östling R, Wang JT-y, Chou EY, & Camerer CF (2011) Testing Game Theory in the Field: Swedish LUPI Lottery Games. *American Economic Journal: Microeconomics* 3(3):1-33.

26. Hampton, AN, Bossaerts, P & O'Doherty, JP. Neural correlates of mentalizing-related computations during strategic interactions in humans. *Proc Natl Acad Sci USA* **105,** 6741-6746 (2008)

27. Davidson, R & Duclos, J-Y. Testing for restricted stochastic dominance. *Econometric Reviews*, 32(1):84–125, 2013

28. Lee D, Conroy ML, McGreevy BP, Barraclough DJ (2004) Reinforcement learning and decision making in monkeys during a competitive game. *Cognitive Brain Research*, 22(1), 45-58

29. Tanaka, M. & Yamamoto, S. Token transfer between mother and offspring chimpanzees (Pan troglodytes): mother-offspring interaction in a competitive situation. *Anim Cogn*. **12,** S19-26 (2009)

30. Yamamoto, S. & Tanaka, M. Do chimpanzees (Pan troglodytes) spontaneously take turns in a reciprocal cooperation task? *J Comp Psychol*. **123,** 242-249 (2009)

31. Martin, C.F., Biro, D. & Matsuzawa, T. Chimpanzees' use of conspecific cues in matching-to-sample tasks: public information use in a fully automated testing environment. *Anim Cogn*. 14, 893-902 (2011)
